# Transcriptome-Based Identification of the Muscle Tissue-Specific Expression Gene *CKM* and Its Regulation of Proliferation, Apoptosis and Differentiation in Chicken Primary Myoblasts

**DOI:** 10.3390/ani13142316

**Published:** 2023-07-14

**Authors:** Bingjie Chen, Yanxing Wang, Dan Hou, Yushi Zhang, Bochun Zhang, Yufang Niu, Haigang Ji, Yadong Tian, Xiaojun Liu, Xiangtao Kang, Hanfang Cai, Zhuanjian Li

**Affiliations:** 1 College of Animal Science and Technology, Henan Agricultural University, Zhengzhou 450001, China; cbj980102@163.com (B.C.); 18700258066@163.com (Y.W.); hd15838268239@163.com (D.H.); zys040828@163.com (Y.Z.); zbc3103186904@163.com (B.Z.); 13462940651@163.com (Y.N.); xingkongjhg@163.com (H.J.); ydtian111@163.com (Y.T.); xjliu2008@hotmail.com (X.L.); xtkang2001@263.net (X.K.); 2 Key Laboratory of Livestock and Poultry Resources (Poultry) Evaluation and Utilization, Ministry of Agriculture and Rural Affairs, Zhengzhou 450001, China

**Keywords:** *CKM*, chicken primary myoblasts, proliferation, apoptosis, differentiation, RNA sequencing

## Abstract

**Simple Summary:**

The study of skeletal muscle growth and development has historically been viewed as an entry point and as a breakthrough event in the improvement of animal breeding in the livestock industry. Muscle is the major constituent of any meat product, and its growth performance and the resulting product quality are the core foci of modern breeding. In this study, we found that *creatine kinase M-type-like (CKM)* was specifically highly expressed in chicken muscle tissue. The quantitative real-time PCR (qPCR) results were consistent with the sequencing results. By overexpressing *CKM* and interfering with *CKM* expression in chicken primary myoblasts, we found that *CKM* could inhibit the proliferation and promote the apoptosis and differentiation of chicken primary myoblasts. Moreover, the transcriptome sequencing results after interference with *CKM* expression indicated that *CKM* is involved in chicken skeletal muscle development. These results suggest that *CKM* may play a crucial role in chicken muscle growth and development.

**Abstract:**

Skeletal muscle is an essential tissue in meat-producing animals, and meat-producing traits have been a hot topic in chicken genetic breeding research. Current research shows that *creatine kinase M-type-like* (*CKM*) is one of the most abundant proteins in skeletal muscle and plays an important role in the growth and development of skeletal muscle, but its role in the development of chicken skeletal muscle is still unclear. Via RNA sequencing (RNA-seq), we found that *CKM* was highly expressed in chicken breast muscle tissue. In this study, the expression profile of *CKM* was examined by quantitative real-time PCR (qPCR), and overexpression and RNA interference techniques were used to explore the functions of *CKM* in the proliferation, apoptosis and differentiation of chicken primary myoblasts (CPMs). It was shown that *CKM* was specifically highly expressed in breast muscle and leg muscle and was highly expressed in stage 16 embryonic muscle, while *CKM* inhibited proliferation, promoted the apoptosis and differentiation of CPMs and was involved in regulating chicken myogenesis. Transcriptome sequencing was used to identify genes that were differentially expressed in CPMs after *CKM* disruption, and bioinformatics analysis showed that *CKM* was involved in regulating chicken myogenesis. In summary, *CKM* plays an important role in skeletal muscle development during chicken growth and development.

## 1. Introduction

The proper development of skeletal muscle in meat-producing animals is positively correlated with increased meat production, making it an economically important tissue; in addition, skeletal muscle plays an important role in initiating locomotion, supporting respiration, and maintaining homeostasis in vivo [1]. Skeletal muscle is composed of a series of muscle fibers made up of multinucleated muscle cells and is formed during development by the fusion of several myoblasts into multinucleated myotubes [2]. Therefore, the study of the proliferation and differentiation mechanisms of myoblasts has become a hot topic in scientific research [3]. Muscle development is generally divided into the embryonic and postnatal stages [4]. In the embryonic stage, muscle progenitor cells differentiate and proliferate to form myoblasts that fuse into multinucleated myotubes, which later mature into muscle fibers with contractile properties [5]. Moreover, the deposition of many substances related to the flavor of meat products begins during the embryonic period [6]. The development of muscle after birth depends on the proliferation and differentiation of myoblasts and the function of muscle satellite cells [7]. Among other events, the morphological structure and number of muscle fibers are finalized during the embryonic period, highlighting the need to explore embryonic muscle development in poultry [8]. Recent studies have shown that many genes are involved in the regulation of chicken skeletal muscle growth and development and that understanding these functional genes is important for breeding animals with a high muscle yield and quality [9,10].

Creatine kinase (CK) exhibits isoenzyme-specific subcellular localization at sites of energy production and consumption. Cells and tissues with intermittent high energy needs, such as bone, myocardial, brain, retinal and sperm cells and tissues, require large amounts of immediately available energy, and creatine kinase plays a key role in the cellular energy metabolism in each of these cells [11]. CK is also required for the synthesis of adenosine triphosphate (ATP), which provides energy for muscle contraction. ATP is replenished by the reversible transfer of an N-phosphoryl group from phosphocreatine to adenosine triphosphate (ADP) [12]. CK constitutes a unique family of oligomeric isoforms with tissue-specific expression and isozyme-specific subcellular localization. There are three cytoplasmic isoforms, namely, the ubiquitous brain-type CK isoform (*CK-BB*), muscle-type CK isoform (*CK-MM*, encoded by the *CKM* gene) and *CK-MB* heterodimer [13], and two mitochondrial isoforms, the ubiquitous *CK-MM*, encoded by the *CKM* gene (*Mia-CK*), and *Mib-CK* [14].

In fully differentiated skeletal muscle, *CK-MM* is abundant, along with mitochondrial *Mib-CK* [15]. In chickens, the creatine kinase M-type-like isozyme (LOC107051134, *CK-MM*, *Gallus gallus*; also called *CKM*) is specifically expressed in skeletal muscle. In humans, *CKM* is specific to skeletal muscle and is used as a marker for muscle damage [16], muscular dystrophy [17], infectious diseases [18], acute renal failure, myocardial infarction [19], rheumatoid arthritis [20], and some liver diseases [21]. Previous studies have shown that the ATP synthesis capacity is significantly decreased in the muscle tissue of *CKM*-knockout mice [22]. Xiong et al. found that *CKM* may be related to the differentiation of myoblasts [23].

Many studies have shown that the *CKM* gene plays an important role in the development of skeletal muscle, but its function in chicken myogenesis is unclear. In this study, the potential regulatory gene *CKM*, which is specifically highly expressed in muscle tissues, was identified through the integrated analysis of RNA sequencing (RNA-seq) data from 21 tissues, and the effects of *CKM* on the proliferation, apoptosis and differentiation of chicken myoblasts were investigated using overexpression and interference techniques.

## 2. Materials and Methods

### 2.1. Ethics Approval

In this study, animal experiments and nursing were carried out according to the Regulations for the Administration of Affairs Concerning Experimental Animals (Ministry of Science and Technology, Beijing, China, 2004), and the experimental scheme was approved by the Animal Care and Use Committee (IACUC) of Henan Agricultural University (approval number: 11-0085). We conducted this study in strict accordance with the ARRIVE guidelines.

### 2.2. Sample Collection

A total of 20 fertilized eggs of Arbor Acres (AA) chickens at 11 embryonic ages (E11, the eleventh day after incubation in an incubator) were provided by Henan Poultry Industry Limited Liability Company (Zhengzhou, China) for the cell culture. The breast muscle and leg muscle tissues of E10, E12, E14, E16, 1-week-old and 3-week-old AA chickens were collected, eight in each period. At the same time, the heart, liver, spleen, lung, kidney, gizzard and ileum tissues of eight AA chickens aged 1-week-old were also collected. Our method of euthanasia for chickens is cervical dislocation. All tissue samples were frozen in liquid nitrogen and placed in a −80 °C freezer for later use.

### 2.3. Data Collection

In this study, 169 RNA-seq datasets (Table S1) were collected from 21 chicken tissues (breast muscle, bursa of Fabricius, heart muscle, caecal tonsil, cerebellum, duodenum, gizzard fat, harderian gland, ileum, kidney, left optic lobe, liver, lung, ovary, pancreas, proventriculus, skin, spleen, thymus, thyroid and trachea). These data were downloaded from the NCBI Database Sequence Read Archive database.

### 2.4. Rapid Amplification of cDNA Ends (RACE)

Total RNA was first extracted from 1-day-old AA chicken leg muscle, and subsequently, Rapid amplification of cDNA End(RACE PCR) was performed on the *CKM* gene using the TAKARA SMARTer (TaKaRa, Tokyo, Japan) 5′ and 3′ RACE kits, according to the manufacturer’s instructions. The primer sequences are shown in Table S2.

### 2.5. Plasmid Construction and Small Interfering RNA (siRNA) Synthesis

The coding sequence (CDS) fragment of the *CKM* gene sequence was obtained as described above, and the pcDNA3.1 vector (Promega, Madison, WI, USA) was used to construct the overexpression plasmid by the insertion of the CDS into the *HindIII* and *EcoRI* restriction sites. The siRNA was designed and synthesized by GenePharma (Shanghai, China), and the primer sequences are shown in Table S2.

### 2.6. Isolation, Culture and Transfection of Chicken Primary Myoblasts (CPMs)

CPMs were isolated from the leg muscle of AA chickens at E11, as previously described [24]. CPMs were cultured in high-sugar Dulbecco’s modified Eagle medium (DMEM) (Biological Industries, Israel) containing 15% fetal bovine serum (FBS) (Biological Industries, Israel). Then, the cells were placed in an incubator at 37 °C and 5% CO_2_ and used for subsequent experiments when the cells were fully grown. For the establishment of the induced differentiation model of CPMs, the cell culture medium was changed to a differentiation medium containing 2% horse serum (Biological Industries, Beit HaEmek, Israel) when the cells reached 95% confluence, and the culture was continued. When CPMs were 95% confluent after differentiation, they were transfected with PC3.1-*CKM* and PC3.1 with Lipofectamine 2000 (Invitrogen, Waltham, MA, USA). Alternatively, si-*CKM* and the negative control siRNA (si-NC) were transfected into CPMs at 40% confluence with Lipofectamine 2000 (Invitrogen, Waltham, MA, USA).

### 2.7. RNA Exaction, cDNA Synthesis, and Quantitative Real-Time PCR (qPCR)

The total RNA from cells and tissues was extracted using RNA TRIzol (TaKaRa, Japan), according to the manufacturer’s instructions. The quality of the total extracted RNA was determined by agarose gel electrophoresis, and the concentration of total RNA was measured using a spectrophotometer (Thermo, Waltham, MA, USA). This was followed by cDNA synthesis using a PrimeScriptTM RT Reagent Kit with a gDNA Eraser (TaKaRa, Japan). Then, qPCR was performed using an SYBR Premix Ex Taq kit (TaKaRa, Japan) on an ABI 7500 instrument (Applied Biosystems, Waltham, MA, USA). The 2^−ΔΔCt^ method was used for the relative quantification of genes, using *GAPDH* as the internal reference gene. The primer sequences used for qPCR are shown in Table S2.

### 2.8. Cell Counting Kit-8 (CCK-8) Assay

CPMs were seeded in 96-well cell plates, 10 µL of CCK-8 reagent was added to each well at 10, 22 and 34 h after transfection and incubation was continued in an incubator. After 2 h of incubation, the absorbance values at 450 nm were obtained using a multifunctional microplate reader (BioTek, Winooski, VT, USA) to determine cell viability. Six biological replicates were used for each group.

### 2.9. 5-Ethynyl-2′-Deoxyuridine (EdU) Incorporation Assay

CPMs were cultured in 24-well cell plates. Then, 22 h post-transfection, a prepared 50 nM EdU staining solution was added, and the plates were incubated for 2 h. Subsequent processing was performed according to the instructions of the Cell-Light EdU Apollo567 In Vitro Kit (RiboBio, Guangzhou, China), and, finally, a fluorescence inverted microscope (Olympus, Tokyo, Japan) was used to acquire images in three random fields of view. Then, the data were analyzed using ImageJ software (version 6.0) (NIH, Bethesda, MD, USA).

### 2.10. Flow Cytometry Analyses

Cell cycle analysis: CPMs were cultured using six-well cell plates, and after 24 h of transfection, the CPMs were harvested, fixed with precooled 75% ethanol and subsequently stored overnight at 4 °C. The RNase A:PI working solution was prepared as a staining solution and was added to CPMs at a volume ratio of 1:9 and incubated for 30 min.

Apoptosis analysis: CPMs were seeded in six-well cell plates after 48 h of transfection. CPMs were collected and subsequently processed according to the Annexin V-FITC apoptosis assay kit (Beyotime, Shanghai, China) instructions.

Finally, cells from both of these assays were analyzed by a BD Accuri C6 flow cytometer (BD Biosciences, Franklin Lakes, NJ, USA), with three biological replicates per group.

### 2.11. Immunofluorescence Analysis

CPMs were seeded in 12-well cell plates, and after 48 h of transfection, the cells were fixed for 20 min at 4 °C using a cell fixative (Solarbio, China) and washed twice with PBS (Solarbio, Beijing, China) for 5 min each. Next, 1 mL of 0.1% Triton X-100 was added to each well, the membrane was permeabilized at room temperature for 10 min and the cells were then washed with PBS, as described previously. Goat serum (Bioss, Beijing, China) was then added for 1 h at room temperature, and anti-Desmin or anti-MYHC (DHSB, Iowa City, IA, USA) was then added prior to incubation at 4 °C overnight. After removal, the next day, the cells were washed three times with PBS and then incubated with anti-mouse IgG FITC-conjugated antibodies (Bioss, China) or anti-Cy3 (Proteintech, Wuhan, China) for 2 h at 37 °C in a dark incubator. Finally, three random fields of view were imaged using an inverted fluorescence microscope (Olympus, Japan) and incubated for 3 min. ImageJ software (version 6.0) (NIH, Bethesda, MD, USA) was used for statistical analysis.

### 2.12. Western Blotting (WB)

CPMs were seeded in six-well cell plates, and after 48 h of transfection, proteins were extracted from the CPMs using a cell extraction kit (Bioss, China) and quantified with a bicinchoninic acid (BCA) protein assay kit (Epizyme, Tianjin, China). The proteins were denatured by adding loading buffer at 100 °C for 10 min and were then stored at −20 °C. Electrophoresis was performed on a 10% polyacrylamide gel (Epizyme, China), and the proteins were transferred to a PVDF membrane (Beyotime, China) prior to blocking with 5% skim milk powder (Yili, Hohhot, China) for 1 h. Anti-MYHC (DHSB, USA) and anti-β-actin (Proteintech, China) antibodies were added, and the membrane was incubated overnight at 4 °C. The next day, the membrane was incubated with an HRP-conjugated secondary antibody (Proteintech, China) for 1 h 30 min at room temperature. Finally, an ECL chromogenic kit was used for color development, images were acquired using an Odyssey Fc Imager (version 5.2.5) (LI-COR, Lincoln, NE, USA) and ImageJ software was used for analysis (NIH, Bethesda, MD, USA). β-actin was used as an internal control.

### 2.13. RNA-seq and Differential Expression Genes (DEGs) Analysis

CPMs were isolated, cultured in vitro and transfected with si-*CKM* and si-NC for 48 h; the total RNA was then extracted using TRIzol (TaKaRa, Japan) prior to transcriptome library construction. mRNA was purified from the total RNA by oligo magnetic beads and the mRNA was randomly fragmented by adding fragmentation buffer. First-strand cDNA was synthesized by using six-base random primers and reverse transcriptase; second-strand cDNA was then synthesized by adding buffer, dNTPs, RNase H and DNA polymerase I. The purified double-stranded cDNA was then end-repaired, A-tailed and ligated to sequencing junctions, and then the library fragments were purified. Finally, the cDNA library was enriched by PCR. The library was evaluated on an Agilent 2100 Bioanalyzer (Agilent Technologies, Santa Clara, CA, USA) (effective concentration >2 nM) and sequenced using the Illumina NovaSeq 6000 platform.

Subsequently, quality control was performed on raw reads, using FASTQ to filter low-quality data in order to obtain clean reads. The reference genome *Gallus gallus* 6.0 and paired-end clean reads were mapped to the reference genome using HISAT2 (version 2.1.0). For each transcribed region, the fragments per kilobase million (FPKM) values were calculated using StringTie software (version 2.2.0) to quantify their expression abundance. Differential expression analysis of the samples was performed using DESeq2 (version 1.40.2) and edgeR software (version 2.4), and we identified genes with a false discovery rate (FDR) <0.05 and |log2 fold change| >1 as DEGs. Our analysis of the DEGs associated with 169 transcriptome data downloaded from the NCBI database is the same as above.

### 2.14. Gene Ontology (GO) and Kyoto Encyclopedia of Genes and Genomes (KEGG) Analyses

GO analysis was used to screen for differentially expressed genes to predict potentially affected biological processes and functions. KEGG pathway analysis was used to determine the functions of molecular pathways potentially involving the differentially expressed genes. The statistical significance was set at *p* < 0.05.

### 2.15. Statistical Analysis

All data are expressed as the mean ± standard error of the mean (SEM) values. Student’s *t* test was performed on the data using IBSS SPSS (SPSS for Windows, standard version 24.0; SPSS, Chicago, IL, USA). Differences were considered significant when 0.01 ≤ *p* ≤ 0.05 and highly significant when *p* < 0.01 (* *p* < 0.05; ** *p* < 0.01, *** *p* < 0.001). The calculated data were visualized and analyzed using GraphPad Prism 7 (GraphPad Software, San Diego, CA, USA). All data are presented as the results of three technical and biological replicates.

## 3. Results

### 3.1. Identification and Analysis of Chicken Tissue-Specific Genes

**Figure 1:**
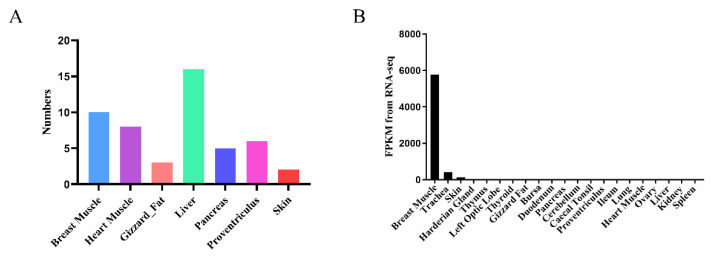
Identification and analysis of chicken tissue-specific genes. (**A**) Number of chicken tissue-specific highly expressed genes (we performed RNA sequencing on 21 tissue types collected from nine 16/17-week-old female J-line chickens); (**B**) Expression levels of *creatine kinase M-type-like (CKM)* in different chicken tissues by RNA sequencing (RNA-seq).

A total of 24,403 genes were detected by integrating and analyzing the collected datasets for tissue-specific high-expression gene screening, with an FPKM value greater than 1000. Statistical analysis revealed the number of genes specifically highly expressed in tissues including the breast muscle, heart muscle, gizzard fat, liver, pancreas, proventriculus and skin (Figure 1A, Table S3). These tissue-specific highly expressed genes are important for identifying gene function and expression characteristics in various tissues [25,26]. As meat production traits are the most economically important traits in broiler production, we focused on genes that are specifically expressed in muscle tissue. We identified a gene called *CKM* (LOC107051134) that was specifically highly expressed in chicken breast muscle tissue (Figure 1B).

### 3.2. Expression Pattern of CKM in Chickens

**Figure 2:**
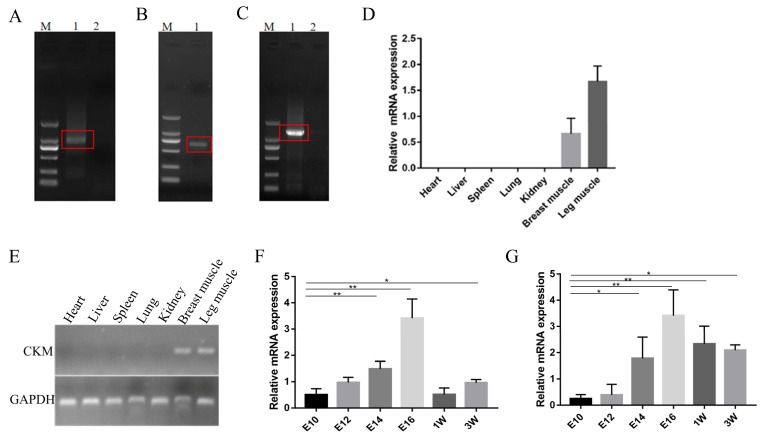
Expression pattern of the *CKM* gene in chickens. (**A**) Results of 3′ Rapid amplification of cDNA End (RACE PCR) amplification of *CKM*; (**B**) Results of 5′ RACE PCR amplification of *CKM*; (**C**) Results of sequence validation of *CKM* after RACE; (**D**) quantitative real-time PCR (qPCR) analysis of *CKM* mRNA expression in various tissues of 1-week-old AA chickens; (**E**) Semiquantitative analysis of *CKM* mRNA expression was performed by gel electrophoresis; (**F**) qPCR analysis of the mRNA expression of *CKM* in breast muscle tissues of E10 to E16, 1-week-old and 3-week-old AA chickens; (**G**) qPCR analysis of the mRNA expression of *CKM* in the leg muscle tissues of E10 to E16, 1-week-old and 3-week-old AA chickens. The values indicated by the bars are the means ± SEM. * *p* < 0.05; ** *p* < 0.01.

Based on the known gene sequence information, amplification was performed using the RACE technique, and a product band of approximately 1.0 kbp in length was found to be amplified by 3′ PCR (Figure 2A), yielding 674 bp of an unknown sequence; the 5′ PCR amplification product had a length of 0.6 kbp (Figure 2B), yielding 395 bp of an unknown sequence. The RACE results confirmed that the length of the PCR amplification product was consistent with the expected length (approximately 1.3 kbp) of size for the target fragment (Figure 2C). The full-length sequence of 1321 bp was obtained by sequencing, and the sequence obtained by 3′ RACE and 5′ RACE was verified to have an A-to-G synonymous mutation at position 381 in the NCBI sequence of *CKM* (Figure S1). NCBI BLAST analysis showed that *CKM* was indeed present in the chicken genome. We further explored the expression pattern of *CKM* in AA chickens by qPCR and semi-quantitative assays, showing that the *CKM* showed specific high expression in the breast and leg muscles of 1-week-old AA chickens (Figure 2D,E). However, it was not expressed or not highly expressed in the heart, liver and spleen (they may exhibit other muscle subtypes of CK), which is consistent with the sequencing data results, further suggesting that the *CKM* may be associated with the growth and development of chicken skeletal muscle. In addition, we found that the expression of *CKM* was gradually upregulated during the development of breast and leg muscles in chicken embryos until E16 reached the peak, while the expression level after hatching showed a downward trend (Figure 2F,G). Therefore, we speculated that *CKM* may be involved in embryonic muscle development.

### 3.3. CKM Inhibits the Proliferation of CPMs

**Figure 3:**
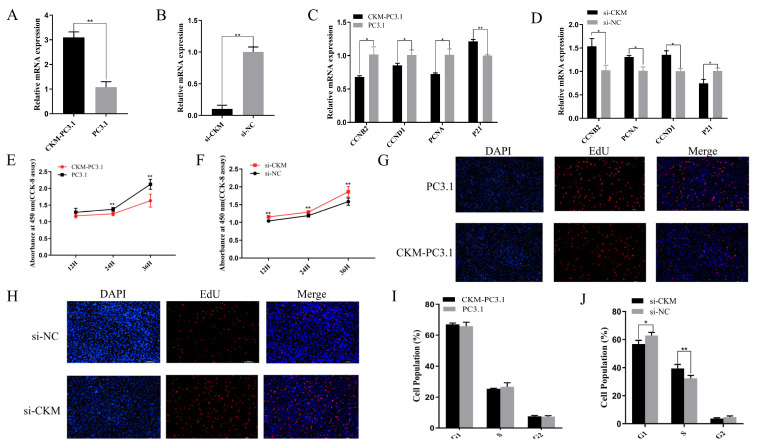
*CKM* inhibits the proliferation of chicken primary myoblasts (CPMs). (**A**) The relative mRNA expression of *CKM* after overexpression, where the *CKM*-PC3.1 group was the treated group with *CKM* overexpression and the PC3.1 group was the control group (empty plasmid without the *CKM* gene sequence); (**B**) The relative mRNA expression of *CKM* after interference, where the si-*CKM* group was the group treated with the *CKM* interference fragment and the si-NC group was the blank control group; (**C**) The mRNA expression levels of *CCNB2*, *CCND1*, *PCNA* and *P21* were measured by qPCR after transfection with the *CKM* overexpression vector; (**D**) The mRNA expression levels of *CCNB2*, *CCND1*, *PCNA* and *P21* were measured by qPCR after transfection with si-*CKM* and si-NC. (**E**) The proliferative activity of CPMs at 12 h, 24 h and 36 h was assessed by a CCK-8 assay after transfection with the *CKM* overexpression vector; (**F**) The proliferative activity of CPMs at 12 h, 24 h and 36 h was assessed by a CCK-8 assay after transfection with si-*CKM* and si-NC; (**G**) EdU incorporation assay after transfection with the *CKM* overexpression vector; EdU (red) fluorescence indicates proliferation and DAPI (blue) fluorescence indicates nuclei. The scale bar represents 100 μm; (**H**) Cells transfected with si-*CKM* and si-NC were subjected to an EdU incorporation assay, with EdU (red) fluorescence indicating proliferation and DAPI (blue) fluorescence indicating nuclei. The scale bar represents 100 μm; (**I**) Cell cycle analysis was performed after transfection with the *CKM* overexpression vector; (**J**) Cell cycle analysis was performed after transfection with si-*CKM* and si-NC. The values indicated by the bars are the means ± SEM. * *p* < 0.05; ** *p* < 0.01.

We first validated the purity of the CPMs isolated from the leg muscles of 11-day-old AA chickens by an Desmin immunofluorescence assay (Figure S2). To further explore the role played by the *CKM* in the proliferation of CPMs, we transfected the *CKM* overexpression vector and control vector, as well as si-*CKM* and si-NC, into CPMs and examined the relative expression of mRNA using qPCR. The results in Figure 3A show that the overexpression of *CKM* resulted in a highly significant difference in mRNA expression levels compared to the control (*p* < 0.01). In addition, the results in Figure 3B show that our designed *CKM* interference fragment resulted in a highly significant reduction in *CKM* gene expression compared to the control group (*p* < 0.01); thus, both the *CKM* overexpression vector and the interference fragment were used for further experiments. Subsequently, we transfected the *CKM* overexpression vector and measured the expression of genes capable of regulating cell cycle-related factors, such as cyclin B2 (*CCNB2*), cyclin D1 (*CCND1*), proliferating cell nuclear antigen (*PCNA*) and cyclin kinase inhibitor (*P21*). The results in Figure 3C show that *CCNB2*, *CCND1*, and *PCNA* expression was significantly downregulated (*p* < 0.05), while *P21* expression showed highly significant upregulation (*p* < 0.01) after the overexpression of *CKM* compared to the control group. In addition, the expression of proliferation-related factors (*CCNB2*, *PCNA*, *CCND1*) was significantly upregulated (*p* < 0.05), and the expression of the cell proliferation factor *P21* was significantly downregulated (*p* < 0.05) after transfection with si-*CKM* compared to that after transfection with si-NC (Figure 3D). In addition, as shown in Figure 3E, the results of the CCK-8 assay showed that the overexpression of *CKM* decreased the viability of myoblasts; the inhibition rate increased with an increasing treatment time, and *CKM* overexpression extremely significantly inhibited the proliferation of myoblasts at 24 h and 36 h (*p* < 0.01) compared to that in the control group. Moreover, the results of the EdU incorporation assay (Figure 3G) indicated that overexpression of the *CKM* gene was able to inhibit the proliferation of myoblasts. In contrast, the CCK-8 and EdU incorporation assay results showed that interference with *CKM* expression significantly promoted the proliferation of myoblasts (Figure 3F,H). In addition, as shown in Figure 3I,J, the cell cycle analysis results showed that, after *CKM* overexpression, the proportion of cells in the G1 phase was increased compared to that in the control group, and the proportion of cells in the S phase was lower than that in the control group, indicating that cell division was inhibited after *CKM* overexpression. The proportion of cells in the G1 phase was significantly higher (*p* < 0.05) than that in the control group. Taken together, these results suggest that *CKM* can inhibit the proliferation and promote the apoptosis of CPMs.

### 3.4. CKM Promotes Apoptosis in CPMs

**Figure 4:**
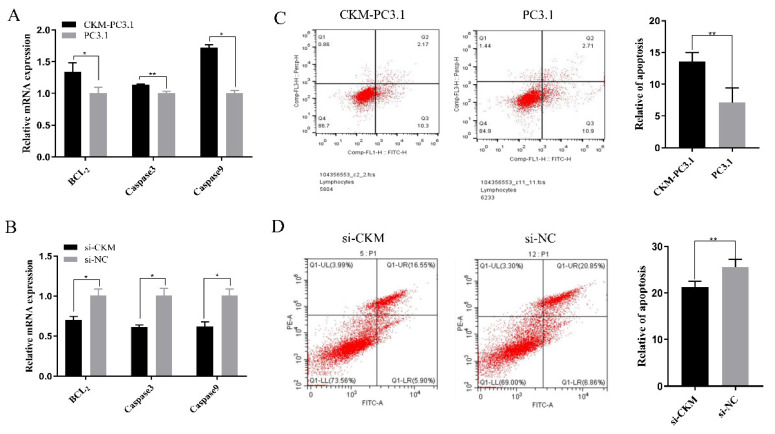
*CKM* inhibits apoptosis in CPMs. (**A**) The mRNA expression levels of *Caspase3* and *Caspase9* by qPCR after transfection with the *CKM* overexpression vector; (**B**) The mRNA expression levels of Caspase3 and Caspase9 were measured by qPCR after transfection with si-*CKM* and si-NC; (**C**) Apoptosis analysis was performed after transfection with the *CKM* overexpression vector; (**D**) Apoptosis analysis was performed after transfection with si-*CKM* and si-NC. The values indicated by the bars are the means ± SEM. * *p* < 0.05; ** *p* < 0.01.

We further examined the effect of *CKM* on apoptosis in CPMs. First, we examined the changes in the relative mRNA expression of the apoptosis-related factor cystatin protease 3 (*Caspase3*) and the apoptosis-related factor cystatin protease 9 (*Caspase9*) by qPCR. The results in Figure 4A show that *Caspase3* expression was very significantly upregulated (*p* < 0.01) and Caspase9 expression was significantly upregulated (*p* < 0.05) after the overexpression of *CKM* compared to the control group. Moreover, the transfection of si-*CKM* significantly downregulated (*p* < 0.05) the expression of the apoptosis-related factors *Caspase3* and *Caspase9* compared with that in the si-NC group (Figure 3B). In addition, as shown in Figure 4C,D, the results of the apoptosis analysis by flow cytometry showed that the number of apoptotic cells was significantly increased after the overexpression of *CKM* compared to that in the control group (*p* < 0.01) and was significantly lower after interference with *CKM* (*p* < 0.01) than that in the control group. In summary, these results suggest that *CKM* can promote apoptosis in CPMs.

### 3.5. CKM Promotes the Differentiation of CPMs

**Figure 5:**
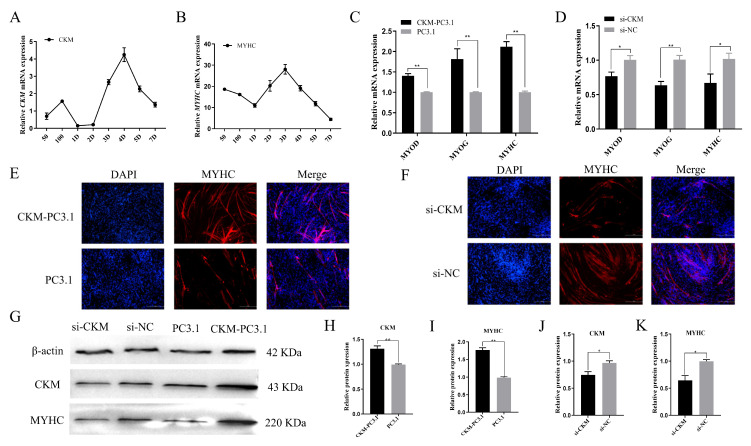
*CKM* promotes the differentiation of CPMs. (**A**) The mRNA expression levels of *CKM* in a model of CPMs proliferation and differentiation; (**B**) The mRNA expression levels of *MYHC* in a model of CPMs proliferation and differentiation; (**C**) The mRNA expression levels of *MYOD*, *MYOG* and *MYHC* were measured by qPCR after transfection with the *CKM* overexpression vector; (**D**) The mRNA expression levels of *MYOD*, *MYOG* and *MYHC* were measured by qPCR after transfection with si-*CKM* and si-NC; (**E**) The differentiation degree of CPMs was determined by MYHC immunofluorescence staining after transfection with the *CKM* overexpression vector (scale bar, 100 μm); (**F**) CPM differentiation levels were detected by MYHC immunofluorescence staining after transfection with si-*CKM* and si-NC (scale bar, 100 μm); (**G**) WB was performed to measure the CKM and MYHC protein expression levels after transfection with the *CKM* overexpression vector and the CKM interference fragment; (**H**,**I**) Quantitative visualization of CKM and MYHC protein expression levels (grayscale values) after transfection with the *CKM* overexpression vector; (**J**,**K**) The protein expression levels of CKM and MYHC after the transfection of si-CKM and si-NC were quantitatively visualized (grayscale values). The values indicated by the bars are the means ± SEM. * *p* < 0.05; ** *p* < 0.01.

To further determine the role of *CKM* in CPMs differentiation, we first used qPCR to measure the mRNA expression levels of *CKM* and myosin heavy chain (*MYHC*) in the CPMs proliferation and differentiation model established earlier. As shown in Figure 5A,B, the overall trends in *CKM* and *MYHC* expression were consistent, first decreasing, then increasing and then decreasing, with the lowest expression on day 1 of differentiation. Both expression levels showed an increasing trend upon cell entry into the redifferentiation stage, indicating that *CKM* is involved in the differentiation of myoblasts. Then, we used qPCR to measure the changes in the mRNA expression of marker genes related to cell differentiation, including a myoblast factor (*MYOD*), myogenin (*MYOG*) and *MYHC*, after transfection with the *CKM* overexpression vector (Figure 5C). The results in Figure 5D show that interference with *CKM* significantly decreased the mRNA expression levels of *MYOD* and *MYHC* (*p* < 0.05) and very significantly decreased the mRNA expression level of *MYOG* (*p* < 0.01). In addition, after the transfection of the *CKM* overexpression vector, we evaluated the differentiation degree of CPMs using *MYHC* immunofluorescence staining (Figure 5E). The results showed that the overexpression of *CKM* significantly promoted the expression of the protein encoded by *MYHC*, thus promoting the differentiation of CPMs. Moreover, the opposite effect was observed by *MYHC* immunofluorescence staining after interference with *CKM* (Figure 5F).

Furthermore, we examined the protein expression levels of *CKM* and *MYHC* by WB. As shown in Figure 5G, the expression of the proteins encoded by both *CKM* and *MYHC* was highly significantly upregulated after the overexpression of *CKM* (*p* < 0.01, Figure 5H,I). In contrast, the levels of the proteins encoded by both *CKM* and *MYHC* were significantly decreased after disrupting the *CKM* expression (*p* < 0.05, Figure 5J,K). At the translational level, the changes in *CKM* expression were consistent with the changes in the expression of the differentiation marker *MYHC*, which more fully shows that the overexpression of *CKM* could promote myoblast differentiation, while interference with *CKM* expression showed the inverse effect. In summary, these results suggest that *CKM* can promote the differentiation of CPMs.

### 3.6. RNA-seq Analysis of Chicken Primary Myoblasts after CKM Interference

**Figure 6:**
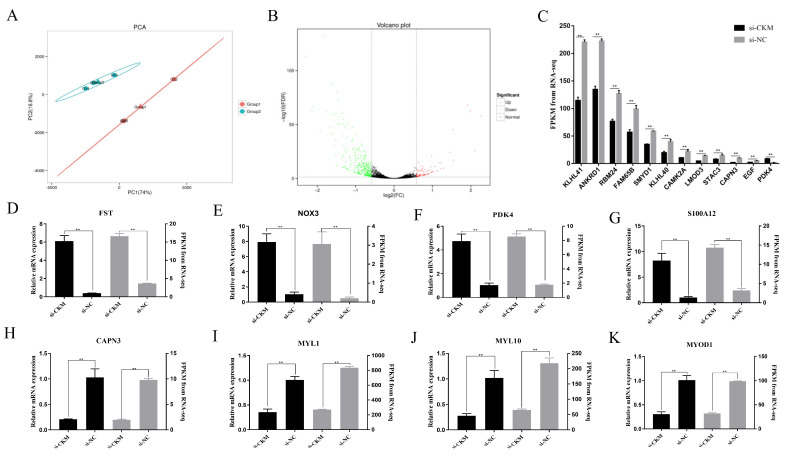
Differentially expressed genes identified by the RNA−seq of CPMs after interference with *CKM* expression. (**A**) PCA of RNA−seq data after transfection with si−*CKM* and si−NC. Group 1 comprised the si−*CKM* treatment group samples (G22, G23, G24); Group 2 comprised the si−NC control group samples (G14, G15, G16); (**B**) Volcano plots of differentially expressed genes in the si−*CKM* and si−NC groups. Green indicates downregulated genes, red indicates upregulated genes and black indicates non-significantly differentially expressed genes; (**C**) The mRNA expression levels of the differentially expressed genes *CAPN3*, *PDK4*, *RBM24*, *SMYD1*, *KLHL41*, *KLHL40*, *STAC3*, *FAM65B*, *LMOD3*, *ANKRD1*, *EGF* and *CAMK2A* were measured by qPCR after interference with *CKM* expression; (**D**–**G**) The mRNA expression levels of the upregulated genes *FST*, *NOX3*, *PDK4* and *S100A2* were measured by qPCR. The abscissa shows the interference treatment group, the left ordinate shows the gene expression changes detected using qPCR and the right ordinate shows the FPKM values determined by sequencing; (**H**–**K**) The mRNA expression levels of the downregulated genes *CAPN3*, *MYL10*, *MYL1* and *MYOD1* were measured by qPCR. The abscissa shows the interference treatment group, the left ordinate shows the gene expression changes detected using qPCR and the right ordinate shows the FPKM values determined by sequencing. The values indicated by the bars are the means ± SEM. ** *p* < 0.01.

To further explore the molecular mechanism of the *CKM* regulation of chicken growth and development, we used transcriptome sequencing technology to compare the differentially expressed genes in primary CPMs treated with si-*CKM* and si-NC by bioinformatic methods. First, the results of the principal component analysis (PCA) are shown in Figure 6A. The two treatment groups can be clearly distinguished, and the si-*CKM* interference group (Group 1) and the si-NC interference control group (Group 2) are clustered together; therefore, the trends in the measured samples remain consistent. This pattern further indicates the reliability of the samples and ensures the accuracy of subsequent tests. There were 439 differentially expressed genes in the si-*CKM* group compared to those of the si-NC group, with 81 upregulated genes and 357 downregulated genes, as shown in the volcano plot (Figure 6B). The results of the GO term analysis of the differentially expressed genes after *CKM* interference showed that the molecular functions of the genes included skeletal muscle fiber adaptation, the positive regulation of sarcomere tissue and myoblast differentiation (Figure S3A). The cellular components related to these genes included the Z-line (a disc-like structure at the junction of two sarcomeres adjacent to myofibrils, which is the location of α-actinin and is connected with fine myofilaments); cardiac myofibrils; myofibrils and other cellular components (Figure S3B). The biological processes included creatine kinase activity, actin binding and calcium-dependent protein kinase activity (Figure S3C). In addition, MAPK signaling pathways, the regulation of the actin cytoskeleton and amino acid metabolism and biosynthesis, which are related to the regulation of skeletal muscle development, were the main KEGG pathways enriched in the differentially expressed genes (Figure S4).

In addition, we analyzed and screened differentially expressed genes associated with important GO terms and KEGG pathways based on our sequencing results and references to other studies on skeletal muscle development. Based on the available literature, we identified highly significantly differentially expressed genes that regulate myoblast development and participate in the muscle growth process, including *CAPN3* [27], *PDK4*, *RBM24* [28], *SMYD1* [29], *KLHL40* [30], *KLHL41* [31], *STAC3* [32], *FAM65B* [33], *LMOD3* [34], *ANKRD1* [35], *EGF* [36] and *CAMK2A* [37] (*p* < 0.01, Figure 6C). Subsequently, we screened the differentially expressed genes with |log2 fold change| ≥1.5 and randomly selected four genes that were upregulated (*FST*, *NOX3*, *PDK4* and *S100A2*) (Figure 6D–G) and four that were downregulated (*CAPN3*, *MYL10*, *MYL1* and *MYOD1*) (Figure 6H–K) for qPCR validation. The results were consistent with the RNA-seq results, demonstrating the accuracy of the sequencing data and showing that the expression of these highly significantly downregulated differentiation-related genes was reduced after interference with *CKM* expression. In conclusion, these results indicate that *CKM* plays an important role in the development of CPMs.

## 4. Discussion

Recently, several studies have been conducted at the transcriptional level in poultry to investigate and analyze the genes and regulatory pathways that influence skeletal muscle growth and development; these studies have helped to reveal genes and pathways associated with muscle development by comparing gene expression profiles for specific traits in various animal populations, thereby facilitating the selection of candidate genes associated with important traits [38]. To date, RNA-seq has been used to discover and study specific genes and pathways for muscle development under different conditions in animals such as chickens [39] and ducks [40]. Wang et al. found that *CKM* may be a key gene involved in the regulation of bovine skeletal muscle development through multi-omics analysis [41]. Bouwman F.G. et al. have shown that a subtype of *CKM* is a new type of fatigue biomarker in horses, which indicates that the change in energy distribution in muscle cells is part of the etiology of the disease [42]. However, there have been fewer studies on skeletal muscle development in chickens. Therefore, in this study, based on the integrated analysis of expression data from different tissues of chickens, we screened for a high expression of *CKM* specifically in muscle tissues and performed qPCR in various tissues of 1-week-old AA chickens, and we found that *CKM* showed specifically high expression in muscle tissues. However, muscle development occurs in two main stages: embryonic and postnatal. Muscle fibers are formed during the embryonic stage, and their number is maintained after birth [43]. We found that *CKM* expression was the highest at E16 during the development of breast muscle and leg muscle. At the same time, studies have shown that *CKM* is involved in the gene regulation of skeletal muscle development and has an important impact on muscle differentiation [41,44]. In addition, *CKM* levels are altered in response to muscle damage and are therefore used as a marker to identify muscle integrity [16]. This finding led to the hypothesis that *CKM* is involved in skeletal muscle development and may play a major role in muscle development during the embryonic p stage in chickens.

Numerous studies have shown that RNA overexpression interference can be used as a means to manipulate gene expression in experiments and investigate gene function on a genome-wide scale [45]. Recently, it has been found that the expression of genes associated with muscle development can influence the development of muscle and that the degree of active gene expression reflects the progress of muscle development [46]. Examples of such genes include *CCNB2* [47], *CCND1* [48], *PCNA* [49], *P21* [50], *Caspase3* [51,52] and *Caspase9* [53], which instruct myoblasts to exit the cell cycle, express muscle-specific genes and block the expression of other cell- or tissue-specific genes. Therefore, in this study, by overexpressing and interfering with *CKM* gene expression, we found that *CKM* could significantly promote the downregulation of proliferation-related genes (*CCNB2*, *CCND1* and *PCNA*), promote the upregulation of the antiproliferative gene *P21*, promote the regulation of apoptosis-related genes (*Caspase3* and *Caspase9*), reduce cell viability, decrease the proportion of cells entering the S phase and decrease the cell proliferation rate. In summary, we found that *CKM* can inhibit myoblast proliferation and promote myoblast apoptosis.

Skeletal muscle differentiation is a multistep process in which myoblasts fuse to give rise to muscle fibers, which are tubular multinucleated fibers containing the contractile machinery necessary for muscle contraction [8]. One study revealed that porcine *MYHC* IIA/X-AS stimulates skeletal satellite cells to exit the cell cycle to promote fusion with myotubes [54]. Therefore, to further investigate the role of *CKM* in the differentiation of chicken myoblasts, we first analyzed the expression pattern of *MYHC* and *CKM* during myoblast differentiation. We showed that *MYHC* expression in myoblasts tended to increase and then decrease with time after the initiation of differentiation and that the changes in *CKM* expression during the differentiation period were consistent with those in the expression of *MYHC*, the differentiation marker gene. In addition, the expression levels of differentiation-related marker genes (*MYOD*, *MYOG* and *MYHC*) in CPMs showed a highly significant increase after the overexpression of *CKM*, with the opposite result observed after the interference of *CKM*. This result was also confirmed by the detection of MYHC protein expression levels. Myogenesis is the differentiation process that drives the formation of multinucleated myotubes. However, cell proliferation and phenotypic differentiation are mutually exclusive events [55]. Initially, myoblasts fuse to form small nascent myotubes, which subsequently fuse with other myoblasts to form large, mature myotubes [56]. Therefore, the detection of the differentiation status of myoblasts by MYHC immunofluorescence showed a significant increase in the number of myotubes formed after the overexpression of *CKM*, with the inverse result after interference with *CKM*. It has been shown that the *CKM* promoter has transcriptional initiation activity only during the induced differentiation of myoblasts, and it is speculated that *CKM* is associated with myoblast differentiation [23], which is consistent with our findings. In summary, *CKM* can promote the differentiation of myoblasts, providing new evidence for further revealing the regulatory mechanism of muscle development and mass increase.

To elucidate the molecular regulatory mechanism of *CKM* function in myoblasts, we performed transcriptome sequencing on chicken myoblasts with disrupted *CKM* expression, endeavoring to provide useful references for studies on muscle growth and development in livestock. The results showed that 438 genes were differentially expressed, of which 81 were upregulated and 357 were downregulated. Interestingly, as we expected, *CKM* was one of the top five downregulated genes. Moreover, the sequencing results were examined using qPCR and revealed that the expression levels of *NOX3*, *PDK4*, *FST* and *S100A12* were significantly higher in the interference *CKM* group compared to those in the control group, while the expression levels of *CAPN3*, *MYL10*, *MYL1* and *MYOD1* were reduced, consistent with and indicating the reliability of the sequencing results. In addition, we performed GO enrichment analysis of these differentially expressed genes and found that they were enriched mainly in skeletal muscle fiber development, the regulation of myoblast differentiation, creatine kinase activity and other processes. In addition, KEGG pathway analysis showed that the differentially expressed genes were enriched mainly in the MAPK signaling pathway [57,58], the calcium signaling pathway [59], the regulation of actin cytoskeleton, the amino acid metabolism, biosynthesis and other related signaling pathways. In conclusion, these differentially expressed genes were enriched mainly in pathways related to skeletal muscle development, thus suggesting that *CKM* may play an important role in skeletal muscle development.

## 5. Conclusions

In summary, we identified a gene, *CKM*, that was specifically highly expressed in chicken muscle tissue. *CKM* can inhibit the proliferation of CPMs and promote their apoptosis and differentiation. The transcriptome sequencing results showed that interference with the *CKM* gene identified 12 genes that may regulate the development of CPMs and participate in the muscle growth process with significant differential expression; these differentially expressed genes were enriched mainly in pathways related to skeletal muscle development. These results suggest that *CKM* plays an important role in chicken muscle growth and development.

## Data Availability

All data in this study are included in the article content as well as in the Supplementary Materials. Transcriptome data have been uploaded to the NCBI database sequence read archive, accession number PRJNA993231.

## Supplementary Materials

The following supporting information can be downloaded at: https://www.mdpi.com/article/10.3390/ani13142316/s1, Figure S1: *CKM* gene sequence alignment; Figure S2: Identification of CPMs; Figure S3: GO-annotated categorical statistics of differentially expressed genes after the transfection of si-*CKM* and si-NC; Figure S4: Scatter plot of the KEGG pathway enrichment of differentially expressed genes after transfection with si-*CKM* and si-NC; Table S1: Information on 169 RNA sequencing datasets from 21 chicken tissues; Table S2: Primers list; Table S3: Chicken tissue-specific highly expressed genes.
