# Supplementary material for: Transcriptome-Based Identification of the Muscle Tissue-Specific Expression Gene CKM and Its Regulation of Proliferation, Apoptosis and Differentiation in Chicken Primary Myoblasts
Source: Animals (Basel). 2023 Jul 14;13(14):2316. doi: 10.3390/ani13142316 (PMC10376263; doi:10.3390/ani13142316)
Supplement: Supplementary file 1 [file animals-13-02316-s001.zip › Figures S1-S4.pdf]

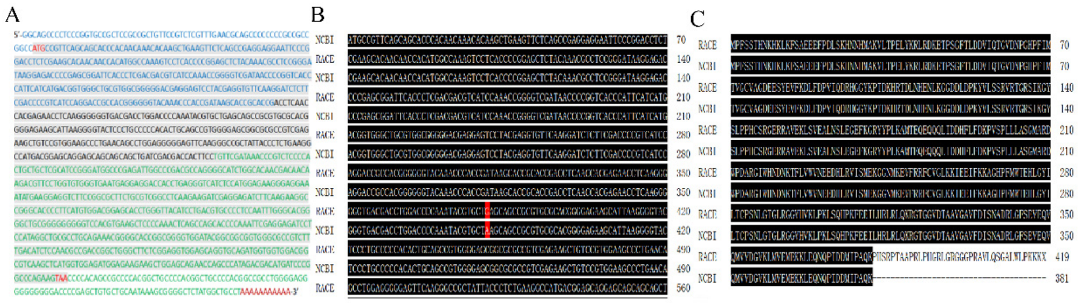

Figure S1. *CKM* gene sequence alignment. (A) Sequence full-length results from RACE of the *CKM* gene. The blue marker sequence is the 5' RACE result, the green marker sequence is the 3' RACE result, and the shaded marker sequence is the CDS region sequence. (B) Sequence alignment results of *CKM* gene. (C) Amino acid sequence alignment results of *CKM* gene.

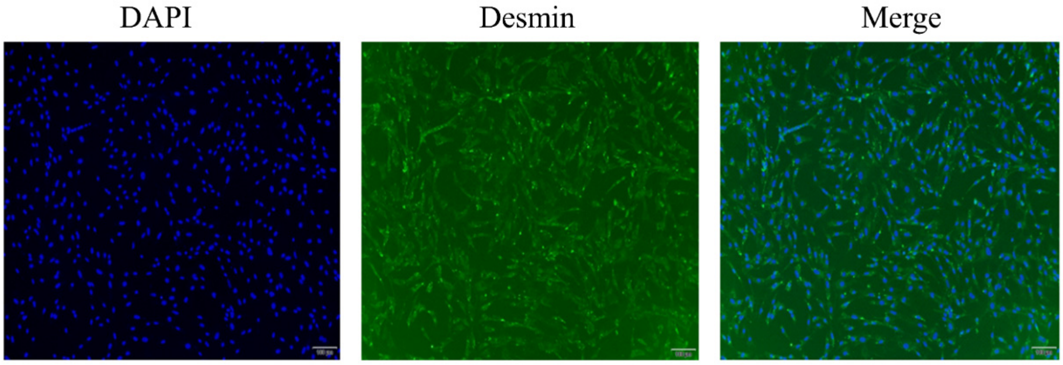

Figure S2. Identification of CPMs. (A) Immunofluorescence detection of Desmin in CPMs. Desmin: myosin specific protein desmin staining; DAPI: staining of nuclei; Merge: merge staining of Desmin and DAPI.

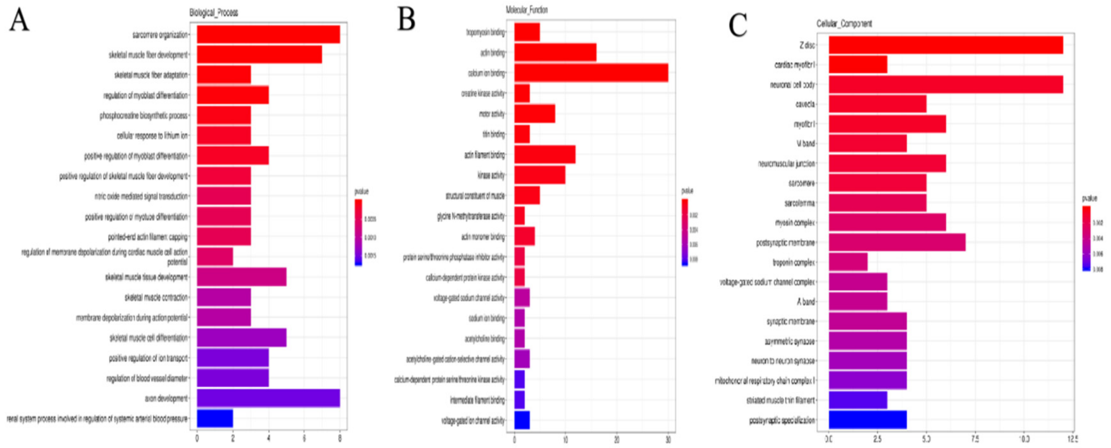

Figure S3. GO annotated categorical statistics of differentially expressed genes after transfection of si-*CKM* and si-NC. (A) Statistical results of GO classification of differentially expressed genes for biological processes. (B) Statistical results of GO classification of differentially expressed genes for molecular function. (C) Results of GO classification of differentially expressed genes in cell components. The horizontal coordinate is the GO classification, the left vertical coordinate is the percentage of the number of genes, and the right vertical coordinate is the number of genes.

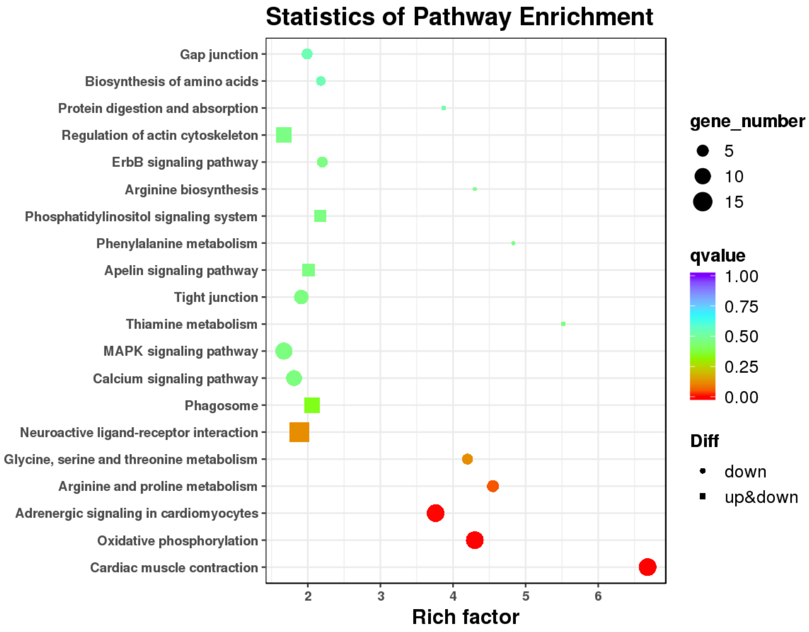

Figure S4. Scatter plot of KEGG pathway enrichment of differentially expressed genes after transfection with si-*CKM* and si-NC.
