# Supplementary material for: Transcriptome-Based Identification of the Muscle Tissue-Specific Expression Gene CKM and Its Regulation of Proliferation, Apoptosis and Differentiation in Chicken Primary Myoblasts
Source: Animals (Basel). 2023 Jul 14;13(14):2316. doi: 10.3390/ani13142316 (PMC10376263; doi:10.3390/ani13142316)
Supplement: Supplementary file 1 [file animals-13-02316-s001.zip › Table S2.pdf]

Supplementary Table S2. Primers list.

Target Primer	Sequence（5'-3'）	Usage
CKM F1	ACCTCAACCACGAGAACCTCAAGG	Target gene sequence verification
CKM R1	GGAAGTGGTCGTCGATCAGCTGCTG	
CKM F2	TGCGCACGGGGAGAAGCATTAAGG	3′ RACE
CKM	CAGCCAGCACCCCAAATTCG	Sequence analysis
CKM R2	TCTACAGACATTGTAGCAATCTAC	5′ RACE
CKM F3	CGCTGTTCCGTCTCGTTTGAACGC	Sequence verification after RACE
CKM R3	TAGAGCCCCGCTTTATTGCAGCAC	
CKM-F4	ATGCCGTTCAGCAGCACCCACAA	Full-length cloning
CKM-R4	CTTCTGGGCCGGGATCATGTCG	
CKM (HindIII) F5	CCCAAGCTTATGCCGTTCAGCAGCAC CCACAA	Double digestion
CKM (BamH) R5	CGCGGATCCCTTCTGGGCCGGGATCA TGTCG	
M13 F	CGCCAGGGTTTTCCCAGTCACGAC	PCR
M13 R	AGCGGATAACAATTTCACACAGGA	
si-*CKM* S	CCGUCUUUGACAUCUCCAATT	Interference fragment
si-*CKM* AS	UUGGAGAUGUCAAAGACGGTT	
si-NC S	UUCUCCGAACGUGUCACGUTT	
si-NC AS	ACGUGACACGUUCGGAGAATT	
CCNB2 F	CCTCTTCCACTTCACTTCT	real-time fluorescence quantitative PCR
CCNB2 R	CTTTGTACCCCACTTATCA	
CCND1 F	CAGAAGTGCGAAGAGGAAGT	
CCND1 R	CTGATGGAGTTGTCGGTGTA	
PCNA F	AGCACCAAATCAGGAAAAG	
PCNA R	GCACAGGAGATGACAACAG	
P21 F	GAAGAGTTGTCCACGATAAGC	
P21 R	TTCCAGTCCTCCTCAGTCC	
Caspase3 F	GGCTACTACTCCTGGAGGA	
Caspase3 F	ACACAATGCATGGAATCTG	
Caspase9 F	GGAGGAGAACAAAAGGACC	
Caspase9 F	CTGGAAAAGTTGAATAGGA	
CKM F5	ACCGACCTCAACCACGAGAA	
CKM R5	AACAGGAAGTGGTCGTCGAT	
GAPDH F	GAACATCATCCCAGCGTCCA	
GAPDH R	CGGCAGGTCAGGTCAACAAC	

Abbreviation: F and R refer to the forward and reverse primers, respectively; S represents the sense chain is the upstream primer, AS represents the antisense chain is the downstream primer.
